# Supplementary material for: A Likelihood Approach for Real-Time Calibration of Stochastic Compartmental Epidemic Models
Source: PLoS Comput Biol. 2017 Jan 17;13(1):e1005257. doi: 10.1371/journal.pcbi.1005257 (PMC5240920; doi:10.1371/journal.pcbi.1005257)
Supplement: S1 File — (TAR.GZ) [file pcbi.1005257.s014.tar.gz › HSPH_Online-SI-Revision/PF08/n10-mild/n10-mild_PF2_table4.pdf]

1	newly 3 weeks spec	153.	230.74	{211., 250.}	50.8105	0.177435	newly 3 weeks cumul	425.	731.05	{686., 769.}	72.0118	0.235147	newly 5 weeks spec	103.	195.53	{172., 218.}	89.835	0.276499	newly 5 weeks cumul	653.	1140.26	{1072., 1197.}	74.6187	0.241707	newly 46 weeks cumul	1470.	2660.53	{2481., 2827.}	80.9884	0.257059
2	newly 3 weeks spec	110.	310.72	{237., 397.}	182.473	0.443638	newly 3 weeks cumul	464.	1023.23	{815., 1248.}	120.524	0.338279	newly 5 weeks spec	57.	239.88	{174., 308.}	320.842	0.612407	newly 5 weeks cumul	610.	1531.44	{1184., 1917.}	151.056	0.393457	newly 51 weeks cumul	742.	2806.64	{1930., 3738.}	278.253	0.564894
3	newly 3 weeks spec	211.	434.45	{347., 528.}	105.9	0.308372	newly 3 weeks cumul	768.	1431.27	{1182., 1705.}	86.3633	0.266904	newly 5 weeks spec	97.	334.85	{245., 415.}	245.206	0.528705	newly 5 weeks cumul	973.	2148.91	{1696., 2587.}	120.854	0.339816	newly 54 weeks cumul	1157.	3753.92	{2628., 4758.}	224.453	0.501864
4	newly 3 weeks spec	113.	337.23	{281., 377.}	198.434	0.471813	newly 3 weeks cumul	452.	1103.54	{956., 1231.}	144.146	0.385013	newly 5 weeks spec	81.	262.78	{210., 302.}	224.42	0.506526	newly 5 weeks cumul	619.	1666.82	{1427., 1866.}	169.276	0.42751	newly 49 weeks cumul	827.	3179.96	{2516., 3584.}	284.518	0.580922
5	newly 3 weeks spec	93.	34.14	{24., 41.}	63.2903	0.442568	newly 3 weeks cumul	276.	119.06	{109., 129.}	56.8623	0.366168	newly 5 weeks spec	92.	25.72	{17., 34.}	72.0435	0.5659	newly 5 weeks cumul	472.	174.37	{159., 194.}	63.0572	0.433543	newly 29 weeks cumul	1568.	338.8	{289., 385.}	78.3929	0.668313
6	newly 3 weeks spec	108.	394.15	{340., 458.}	264.954	0.558888	newly 3 weeks cumul	406.	1204.75	{1070., 1404.}	196.736	0.469574	newly 5 weeks spec	80.	342.35	{267., 410.}	327.938	0.625211	newly 5 weeks cumul	611.	1920.04	{1663., 2255.}	214.245	0.494265	newly 55 weeks cumul	829.	4180.52	{3130., 4935.}	404.285	0.696341
7	newly 3 weeks spec	89.	315.58	{262., 359.}	254.584	0.546529	newly 3 weeks cumul	391.	981.02	{824., 1102.}	150.9	0.397212	newly 5 weeks spec	87.	269.45	{208., 322.}	209.713	0.485894	newly 5 weeks cumul	552.	1546.32	{1310., 1746.}	180.13	0.444741	newly 51 weeks cumul	801.	3499.16	{2578., 4175.}	336.849	0.633532
8	newly 3 weeks spec	197.	237.83	{212., 263.}	20.8071	0.0807593	newly 3 weeks cumul	615.	762.38	{721., 804.}	23.9642	0.0928915	newly 5 weeks spec	164.	199.05	{179., 221.}	21.5915	0.0834929	newly 5 weeks cumul	920.	1178.53	{1115., 1240.}	28.1011	0.107224	newly 46 weeks cumul	2034.	2649.52	{2436., 2847.}	30.2616	0.114037
9	newly 3 weeks spec	116.	376.06	{319., 416.}	224.19	0.508297	newly 3 weeks cumul	592.	1222.37	{1043., 1319.}	106.481	0.313301	newly 5 weeks spec	85.	299.5	{246., 340.}	252.353	0.543652	newly 5 weeks cumul	760.	1861.3	{1556., 2008.}	144.908	0.387244	newly 50 weeks cumul	872.	3609.69	{3031., 4011.}	313.955	0.613817
10	newly 3 weeks spec	91.	251.26	{200., 281.}	176.11	0.438068	newly 3 weeks cumul	345.	795.72	{673., 857.}	130.643	0.360973	newly 5 weeks spec	65.	211.25	{175., 241.}	225.	0.507715	newly 5 weeks cumul	482.	1237.6	{1044., 1340.}	156.763	0.407248	newly 47 weeks cumul	1027.	2836.6	{2440., 3130.}	176.203	0.437317
11	newly 3 weeks spec	165.	286.75	{243., 334.}	73.7879	0.236551	newly 3 weeks cumul	495.	868.87	{778., 989.}	75.5293	0.242136	newly 5 weeks spec	56.	260.47	{203., 307.}	365.125	0.662123	newly 5 weeks cumul	648.	1405.69	{1229., 1615.}	116.927	0.333548	newly 52 weeks cumul	801.	3703.74	{2616., 4492.}	362.39	0.656523
12	newly 3 weeks spec	53.	467.45	{429., 507.}	781.981	0.944632	newly 3 weeks cumul	224.	1618.92	{1536., 1704.}	622.732	0.858637	newly 5 weeks spec	9.	327.73	{291., 360.}	3541.44	1.55991	newly 5 weeks cumul	264.	2345.44	{2237., 2459.}	788.424	0.948287	newly 54 weeks cumul	276.	3522.74	{3341., 3676.}	1176.36	1.10555
13	newly 3 weeks spec	135.	259.63	{226., 288.}	92.3185	0.282003	newly 3 weeks cumul	528.	856.2	{767., 936.}	62.1591	0.208617	newly 5 weeks spec	51.	209.75	{183., 239.}	311.275	0.611405	newly 5 weeks cumul	676.	1302.78	{1189., 1412.}	92.7189	0.283723	newly 46 weeks cumul	774.	2625.74	{2381., 2825.}	239.243	0.52917
14	newly 3 weeks spec	150.	258.24	{229., 285.}	72.16	0.234004	newly 3 weeks cumul	578.	832.32	{767., 896.}	44.	0.157238	newly 5 weeks spec	106.	213.72	{184., 241.}	101.623	0.301746	newly 5 weeks cumul	857.	1282.74	{1175., 1394.}	49.6779	0.173912	newly 47 weeks cumul	1338.	2783.38	{2514., 3047.}	108.025	0.316016
15	newly 3 weeks spec	208.	287.16	{259., 312.}	38.0577	0.138808	newly 3 weeks cumul	656.	885.52	{825., 949.}	34.9878	0.12971	newly 5 weeks spec	192.	254.18	{230., 279.}	32.3854	0.120411	newly 5 weeks cumul	1065.	1410.75	{1334., 1491.}	32.4648	0.121615	newly 49 weeks cumul	2326.	3491.37	{3238., 3722.}	50.1019	0.175712
16	newly 3 weeks spec	196.	341.58	{275., 408.}	74.2755	0.236109	newly 3 weeks cumul	619.	1096.82	{890., 1267.}	77.1922	0.244858	newly 5 weeks spec	104.	279.66	{208., 341.}	168.904	0.42227	newly 5 weeks cumul	881.	1685.69	{1386., 1966.}	91.3383	0.277797	newly 50 weeks cumul	1128.	3413.21	{2558., 4191.}	202.59	0.473213
17	newly 3 weeks spec	103.	243.92	{222., 267.}	136.816	0.373287	newly 3 weeks cumul	286.	765.27	{724., 806.}	167.577	0.427056	newly 5 weeks spec	112.	213.49	{191., 233.}	90.6161	0.278833	newly 5 weeks cumul	495.	1207.8	{1142., 1264.}	144.	0.387042	newly 47 weeks cumul	2645.	2954.67	{2770., 3153.}	11.738	0.0477491
18	newly 3 weeks spec	134.	253.81	{226., 276.}	89.4104	0.276183	newly 3 weeks cumul	498.	798.45	{748., 842.}	60.3313	0.204515	newly 5 weeks spec	79.	222.27	{198., 241.}	181.354	0.448175	newly 5 weeks cumul	686.	1259.54	{1193., 1333.}	83.6064	0.263548	newly 47 weeks cumul	921.	2978.94	{2804., 3134.}	223.446	0.509422
19	newly 3 weeks spec	74.	281.38	{223., 334.}	280.243	0.575848	newly 3 weeks cumul	333.	930.93	{779., 1061.}	179.559	0.443335	newly 5 weeks spec	49.	222.93	{178., 258.}	354.959	0.65283	newly 5 weeks cumul	449.	1407.95	{1199., 1590.}	213.575	0.493106	newly 50 weeks cumul	666.	2720.79	{2185., 3303.}	308.527	0.605152
20	newly 3 weeks spec	165.	305.77	{235., 383.}	85.3152	0.261596	newly 3 weeks cumul	661.	983.94	{804., 1170.}	48.8563	0.168383	newly 5 weeks spec	141.	248.3	{178., 307.}	76.1135	0.237396	newly 5 weeks cumul	964.	1509.37	{1204., 1752.}	56.5737	0.189861	newly 51 weeks cumul	1374.	2986.94	{2027., 3711.}	117.39	0.328046
21	newly 3 weeks spec	122.	304.12	{254., 368.}	149.279	0.392386	newly 3 weeks cumul	466.	1007.82	{873., 1152.}	116.27	0.332179	newly 5 weeks spec	62.	238.31	{192., 292.}	284.371	0.579038	newly 5 weeks cumul	595.	1518.42	{1314., 1785.}	155.197	0.403752	newly 51 weeks cumul	738.	2890.05	{2249., 3707.}	291.606	0.585466
22	newly 3 weeks spec	145.	242.87	{216., 266.}	67.4966	0.222707	newly 3 weeks cumul	458.	774.46	{714., 822.}	69.0961	0.227526	newly 5 weeks spec	86.	208.62	{183., 236.}	142.581	0.383013	newly 5 weeks cumul	631.	1208.66	{1137., 1289.}	91.5468	0.281704	newly 47 weeks cumul	1216.	2802.68	{2565., 3045.}	130.484	0.361449
23	newly 3 weeks spec	51.	339.05	{286., 396.}	564.804	0.816738	newly 3 weeks cumul	384.	1086.64	{934., 1250.}	182.979	0.44799	newly 5 weeks spec	14.	280.42	{216., 346.}	1903.	1.29217	newly 5 weeks cumul	436.	1680.08	{1436., 1962.}	285.339	0.581197	newly 52 weeks cumul	442.	3397.19	{2742., 3993.}	668.595	0.875041
24	newly 3 weeks spec	109.	276.1	{241., 310.}	153.303	0.401111	newly 3 weeks cumul	313.	862.	{754., 933.}	175.399	0.438519	newly 5 weeks spec	56.	234.92	{196., 261.}	319.5	0.619736	newly 5 weeks cumul	432.	1354.5	{1176., 1475.}	213.542	0.494683	newly 48 weeks cumul	566.	3218.68	{2665., 3588.}	468.671	0.751757
25	newly 3 weeks spec	134.	442.32	{391., 506.}	230.09	0.516068	newly 3 weeks cumul	550.	1318.67	{1179., 1459.}	139.758	0.377881	newly 5 weeks spec	58.	400.31	{328., 475.}	590.19	0.834709	newly 5 weeks cumul	716.	2143.71	{1886., 2439.}	199.401	0.474233	newly 55 weeks cumul	798.	4740.33	{3846., 5816.}	494.026	0.769313
26	newly 3 weeks spec	59.	238.03	{204., 266.}	303.441	0.603753	newly 3 weeks cumul	261.	760.62	{708., 808.}	191.425	0.463718	newly 5 weeks spec	33.	198.7	{170., 228.}	502.121	0.776829	newly 5 weeks cumul	353.	1175.59	{1083., 1255.}	233.028	0.521548	newly 46 weeks cumul	475.	2613.06	{2412., 2838.}	450.118	0.73908
27	newly 3 weeks spec	87.	307.21	{257., 353.}	253.115	0.545065	newly 3 weeks cumul	397.	990.96	{850., 1104.}	149.612	0.395319	newly 5 weeks spec	24.	243.53	{212., 275.}	914.708	1.00384	newly 5 weeks cumul	477.	1509.18	{1279., 1668.}	216.39	0.498448	newly 48 weeks cumul	499.	3061.13	{2466., 3334.}	513.453	0.784914
28	newly 3 weeks spec	95.	20.42	{14., 26.}	78.5053	0.682001	newly 3 weeks cumul	273.	70.93	{62., 79.}	74.0183	0.587429	newly 5 weeks spec	72.	15.67	{9., 23.}	78.2361	0.682929	newly 5 weeks cumul	428.	104.56	{91., 117.}	75.5701	0.614083	newly 26 weeks cumul	1195.	201.57	{161., 246.}	83.1322	0.778346
29	newly 3 weeks spec	140.	279.14	{227., 325.}	99.3857	0.295181	newly 3 weeks cumul	520.	892.64	{762., 1016.}	71.6615	0.231378	newly 5 weeks spec	86.	226.9	{168., 274.}	163.837	0.412165	newly 5 weeks cumul	706.	1371.29	{1150., 1560.}	94.2337	0.284023	newly 49 weeks cumul	1008.	2847.48	{2055., 3429.}	182.488	0.44086
30	newly 3 weeks spec	99.	261.41	{199., 315.}	164.051	0.414817	newly 3 weeks cumul	453.	851.25	{695., 1017.}	87.9139	0.269214	newly 5 weeks spec	51.	211.47	{148., 266.}	314.647	0.608203	newly 5 weeks cumul	566.	1296.22	{1003., 1538.}	129.014	0.354537	newly 48 weeks cumul	783.	2648.45	{1756., 3270.}	238.244	0.519273
31	newly 3 weeks spec	92.	379.54	{310., 450.}	312.543	0.610636	newly 3 weeks cumul	415.	1147.33	{926., 1361.}	176.465	0.43676	newly 5 weeks spec	49.	343.63	{277., 414.}	601.286	0.839787	newly 5 weeks cumul	552.	1854.04	{1523., 2156.}	235.877	0.521368	newly 55 weeks cumul	668.	4387.37	{3704., 5142.}	556.792	0.813134
32	newly 3 weeks spec	124.	30.31	{22., 38.}	75.5565	0.621436	newly 3 weeks cumul	386.	105.2	{90., 117.}	72.7461	0.566844	newly 5 weeks spec	137.	23.87	{17., 31.}	82.5766	0.769665	newly 5 weeks cumul	648.	155.2	{134., 175.}	76.0494	0.622958	newly 28 weeks cumul	1971.	300.28	{248., 353.}	84.7651	0.821524
33	newly 3 weeks spec	96.	334.17	{283., 373.}	248.094	0.538393	newly 3 weeks cumul	537.	1111.85	{939., 1219.}	107.048	0.313758	newly 5 weeks spec	49.	260.84	{217., 303.}	432.327	0.721804	newly 5 weeks cumul	666.	1672.33	{1412., 1841.}	151.101	0.397464	newly 49 weeks cumul	775.	3149.86	{2549., 3585.}	306.434	0.60539
34	newly 3 weeks spec	217.	279.96	{244., 317.}	29.8157	0.111671	newly 3 weeks cumul	685.	875.15	{784., 979.}	28.0277	0.105808	newly 5 weeks spec	140.	238.14	{208., 266.}	70.3143	0.2281	newly 5 weeks cumul	982.	1370.98	{1234., 1523.}	39.8004	0.143907	newly 49 weeks cumul	1514.	3105.7	{2639., 3484.}	105.155	0.30827
35	newly 3 weeks spec	115.	277.75	{223., 334.}	141.522	0.377732	newly 3 weeks cumul	444.	906.47	{771., 1060.}	104.16	0.306529	newly 5 weeks spec	59.	221.55	{159., 274.}	275.508	0.565661	newly 5 weeks cumul	616.	1376.85	{1130., 1623.}	123.515	0.344966	newly 49 weeks cumul	837.	2723.41	{1882., 3428.}	225.378	0.501731
36	newly 3 weeks spec	124.	328.15	{278., 383.}	164.637	0.419678	newly 3 weeks cumul	489.	1070.87	{966., 1207.}	118.992	0.33828	newly 5 weeks spec	43.	261.1	{204., 316.}	507.209	0.77736	newly 5 weeks cumul	588.	1625.23	{1424., 1887.}	176.4	0.438945	newly 51 weeks cumul	620.	3108.93	{2567., 3738.}	401.44	0.694668
37	newly 3 weeks spec	61.	324.51	{249., 375.}	431.984	0.719619	newly 3 weeks cumul	281.	1015.51	{828., 1152.}	261.391	0.553969	newly 5 weeks spec	9.	278.88	{187., 346.}	2998.67	1.47963	newly 5 weeks cumul	307.	1597.31	{1256., 1778.}	420.296	0.711059	newly 54 weeks cumul	480.	3478.5	{1993., 4413.}	624.688	0.843915
38	newly 3 weeks spec	169.	312.37	{250., 372.}	84.8343	0.2621	newly 3 weeks cumul	582.	992.54	{832., 1123.}	70.5395	0.22877	newly 5 weeks spec	127.	256.16	{198., 305.}	101.701	0.298125	newly 5 weeks cumul	845.	1533.98	{1200., 1718.}	81.5361	0.2554	newly 50 weeks cumul	1549.	3276.19	{2238., 3884.}	111.504	0.317223
39	newly 3 weeks spec	23.	328.95	{268., 380.}	1330.22	1.15179	newly 3 weeks cumul	145.	1075.63	{942., 1217.}	641.814	0.867637	newly 5 weeks spec	14.	264.27	{210., 310.}	1787.64	1.27029	newly 5 weeks cumul	176.	1634.64	{1392., 1830.}	828.773	0.965087	newly 51 weeks cumul	182.	3097.84	{2395., 3672.}	1602.11	1.22551
40	newly 3 weeks spec	69.	16.89	{12., 23.}	75.5217	0.627187	newly 3 weeks cumul	223.	57.81	{50., 66.}	74.0762	0.588716	newly 5 weeks spec	71.	13.05	{8., 18.}	81.6197	0.759833	newly 5 weeks cumul	369.	86.23	{76., 95.}	76.6314	0.632924	newly 24 weeks cumul	1107.	163.26	{129., 195.}	85.252	0.837286
41	newly 3 weeks spec	7.	517.88	{463., 570.}	7298.29	1.86761	newly 3 weeks cumul	98.	1642.43	{1537., 1728.}	1575.95	1.22357	newly 5 weeks spec	0.1	423.61	{349., 477.}	423510.	3.62298	newly 5 weeks cumul	102.	2538.22	{2359., 2700.}	2388.45	1.39493	newly 55 weeks cumul	102.	4730.09	{3949., 5272.}	4537.34	1.66212
42	newly 3 weeks spec	113.	252.87	{224., 278.}	123.779	0.348146	newly 3 weeks cumul	500.	794.54	{721., 852.}	58.908	0.200027	newly 5 weeks spec	90.	216.8	{180., 243.}	140.889	0.378856	newly 5 weeks cumul	679.	1245.71	{1119., 1337.}	83.4624	0.26232	newly 47 weeks cumul	908.	2915.99	{2424., 3237.}	221.144	0.504205
43	newly 3 weeks spec	16.	454.7	{394., 514.}	2741.88	1.45133	newly 3 weeks cumul	172.	1448.49	{1328., 1590.}	742.145	0.924144	newly 5 weeks spec	1.	372.05	{302., 431.}	37105.	2.56653	newly 5 weeks cumul	187.	2231.47	{1989., 2465.}	1093.3	1.07535	newly 53 weeks cumul	187.	4264.13	{3460., 4928.}	2180.28	1.35436
44	newly 3 weeks spec	169.	276.07	{246., 308.}	63.7574	0.21257	newly 3 weeks cumul	606.	868.56	{790., 959.}	43.6304	0.155985	newly 5 weeks spec	127.	235.91	{208., 268.}	85.7559	0.266255	newly 5 weeks cumul	896.	1362.18	{1242., 1499.}	52.2232	0.181134	newly 49 weeks cumul	1535.	3117.2	{2825., 3519.}	103.075	0.305182
45	newly 3 weeks spec	53.	537.76	{480., 605.}	914.642	1.00441	newly 3 weeks cumul	403.	1811.44	{1662., 1982.}	349.489	0.651707	newly 5 weeks spec	9.	380.97	{321., 439.}	4133.	1.62274	newly 5 weeks cumul	435.	2648.73	{2397., 2908.}	508.903	0.78319	newly 56 weeks cumul	452.	4110.45	{3455., 4699.}	809.392	0.955781
46	newly 3 weeks spec	126.	363.85	{297., 443.}	188.77	0.455698	newly 3 weeks cumul	521.	1133.81	{943., 1352.}	117.622	0.333939	newly 5 weeks spec	38.	307.25	{251., 388.}	708.553	0.901503	newly 5 weeks cumul	617.	1781.42	{1493., 2095.}	188.723	0.456705	newly 53 weeks cumul	658.	3724.46	{2774., 4418.}	466.027	0.746355
47	newly 3 weeks spec	195.	430.74	{353., 503.}	120.892	0.339732	newly 3 weeks cumul	736.	1380.16	{1170., 1593.}	87.5217	0.269763	newly 5 weeks spec	94.	342.25	{264., 412.}	264.096	0.554968	newly 5 weeks cumul	971.	2108.88	{1764., 2433.}	117.186	0.333109	newly 53 weeks cumul	1232.	3959.66	{3157., 4761.}	221.401	0.500863
48	newly 3 weeks spec	116.	293.73	{252., 341.}	153.216	0.399679	newly 3 weeks cumul	481.	922.08	{782., 1051.}	91.7006	0.279709	newly 5 weeks spec	50.	248.53	{207., 283.}	397.06	0.692039	newly 5 weeks cumul	630.	1444.52	{1235., 1653.}	129.289	0.357384	newly 50 weeks cumul	758.	3277.92	{2645., 3658.}	332.443	0.630283
49	newly 3 weeks spec	138.	237.97	{218., 261.}	72.442	0.235294	newly 3 weeks cumul	440.	770.8	{731., 814.}	75.1818	0.242959	newly 5 weeks spec	140.	204.38	{179., 232.}	46.1857	0.162677	newly 5 weeks cumul	714.	1195.89	{1131., 1254.}	67.4916	0.223433	newly 46 weeks cumul	1855.	2705.36	{2516., 2902.}	45.8415	0.16277
50	newly 3 weeks spec	69.	242.52	{202., 272.}	251.478	0.543526	newly 3 weeks cumul	243.	783.48	{690., 843.}	222.42	0.507294	newly 5 weeks spec	43.	202.78	{162., 231.}	371.581	0.669251	newly 5 weeks cumul	357.	1208.03	{1023., 1299.}	238.384	0.527955	newly 46 weeks cumul	518.	2662.87	{2092., 2959.}	414.068	0.707867
